# Unique Surgical Issues in the Management of a Giant Retroperitoneal Schwannoma and Brief Review of Literature

**DOI:** 10.1155/2014/781347

**Published:** 2014-03-06

**Authors:** Santhosh Kuriakose, Syam Vikram, Surij Salih, Satheesan Balasubramanian, Nizamudeen Mangalasseri Pareekutty, Sangeetha Nayanar

**Affiliations:** Department of Surgical Oncology, Malabar Cancer Centre, Moozhikkara P.O., Thalassery, Kannur, Kerala 670103, India

## Abstract

Ancient Schwannoma, though benign, can cause diagnostic dilemma because of its clinical presentation and imaging features. We report the management of a giant retroperitoneal schwannoma in a 19-year-old young lady who presented with lower abdominal distension. CT scan reported a large heterogenous lesion in the abdominopelvic retroperitoneum (42 cm × 16 cm × 16 cm) as a malignant tumor. The unique problems we encountered were the enormous size, the location of major part of the tumor in the pelvis, the need for fertility preservation, the external iliac vessels stretching over the tumor making mobilization surgically demanding, and the prospects of neurological deficits. An en bloc resection of schwannoma with common iliac, external iliac and internal iliac veins, internal iliac artery, femoral and obturator nerves, and iliopsoas muscle was done maintaining oncological principles. External iliac artery that was cut to facilitate tumor mobilization was reanastomosed at the end of the procedure. Postoperatively patient had uneventful recovery with patchy sensory loss, foot drop, and quadriceps weakness which was rehabilitated with a foot drop splint and active physiotherapy.

## 1. Introduction

Giant retroperitoneal schwannoma, though benign, can cause diagnostic dilemma because of its clinical presentation and imaging features. In addition, it is a surgical challenge due to its enormous size and proximity to large vessels and other organs in the retroperitoneum. We are presenting a case, probably one of the largest retroperitoneal schwannomas reported in English medical literature, to discuss the unique surgical problems encountered in the management. The importance of proper anatomical localization of vessels, preoperative planning, and optimal involvement of specialists of other subspecialties for effective resection is reiterated.

## 2. Case Presentation

19-year-old postmenarchal young lady presented with lower abdominal distention and neuritic type of pain in the right thigh of two-year duration. The lower abdominal distension was progressively increasing and was associated with an increasing pain in the leg as described. There were no neurological symptoms.

**Figure 1:**
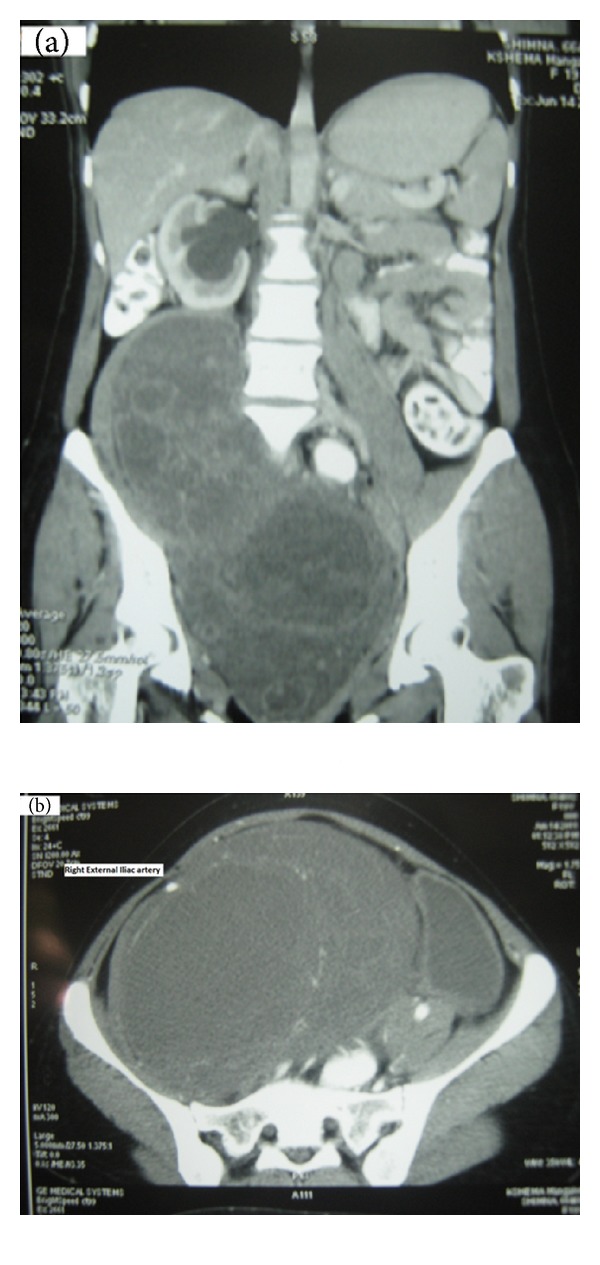
(a) C.T. scan showing large lobulated mass lesion in the pelvic retroperitoneum. (b) Right external iliac artery stretched over the mass.

Clinically she had ECOG performance status 1, antalgic gait, and lower abdominal distension up to umbilicus with a firm to hard fixed mass arising from the pelvis. There were no neurocutaneous markers. Computed tomography study revealed a large lobulated heterogeneously enhancing mass lesion in the pelvic retroperitoneum (craniocaudal measurement 42 cm, anteroposterior measurement 16 cm, and transverse measurement 16 cm) (Figure 1(a)). The mass pushed and displaced right common iliac artery and external iliac artery anterolaterally over the tumor (Figure 1(b)). Moderate hydroureteronephrosis was noted on the right side. Provisional diagnosis of retroperitoneal sarcoma was made on the basis of aforementioned features. In view of its large size with suspicion of resectability, an ultrasound guided fine needle aspiration cytology and core biopsy was done. They were reported as benign nerve sheath tumor. Exploratory laparotomy was undertaken by a midline incision from xiphisternum to symphysis pubis. There was a large lobulated mass (42 cm × 16 cm × 16 cm), encapsulated by the right psoas muscle with majority of tumor in the pelvic retro-peritoneal compartment, with displacement of urinary bladder and uterus to the left side. Tumor extended up to levator ani inferiorly and along the right paraspinal region up to the lower pole of right kidney superiorly. Right common iliac and external iliac arteries with corresponding veins were stretched over the anterolateral aspect of the tumor. The tumor was found splaying the bifurcation of common iliac vein. Ipsilateral ureter was found stretched over the tumor with pressure effect resulting in hydroureteronephrosis. The right femoral and obturator nerves were found involved by the tumor.

**Figure 2:**
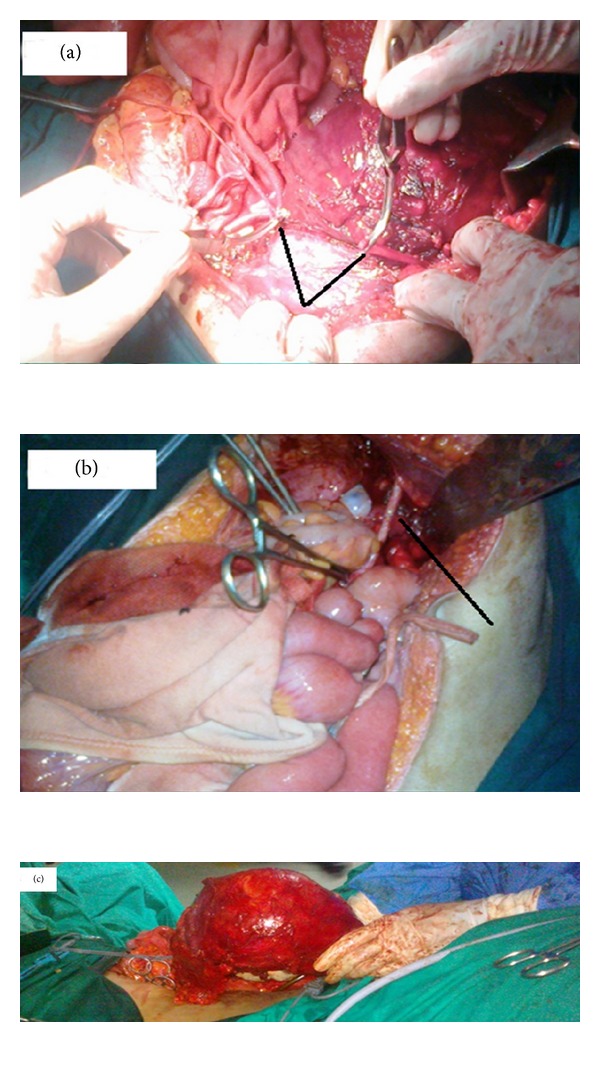
(a) Right external iliac artery cut prior to mobilization and held with bulldog clamps (V arrow showing cut edges of the artery). (b) Right external iliac artery after re-anastomosis (black line showing point of anastomosis). (c) En-bloc radical excision specimen.

**Figure 3:**
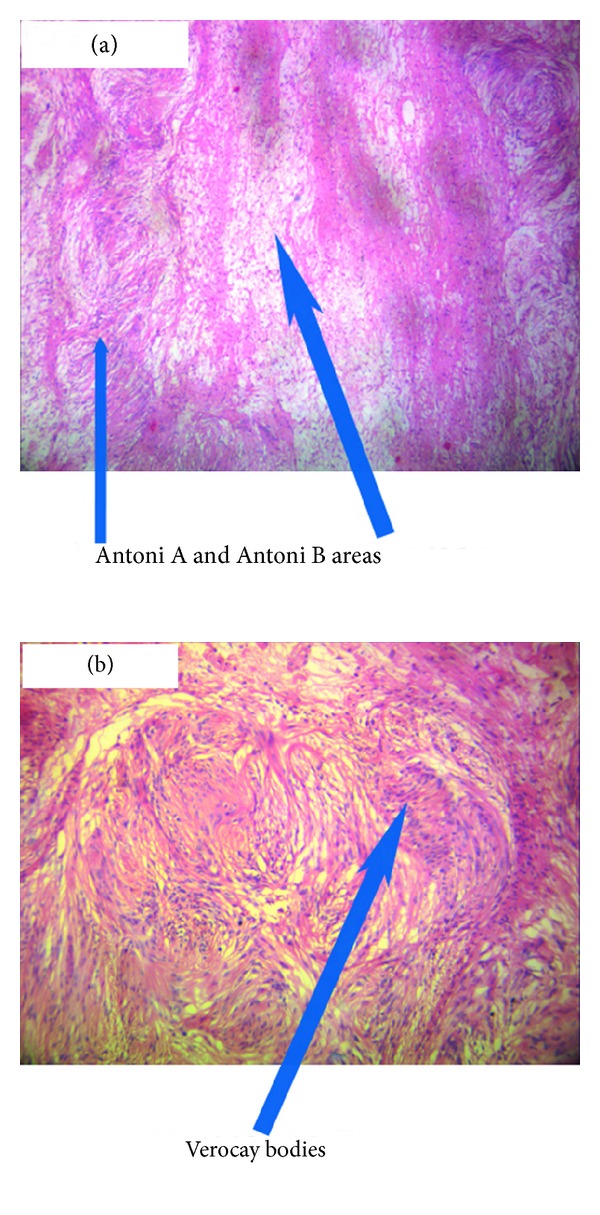
Histopathology of ancient schwannoma showing spindle cells arranged in dense Antoni A bearing Verocay bodies and loose Antoni B patterns.

The external iliac vessels stretching over the mass precluded any mobilization without vascular injury. Hence the external iliac artery was cut at the middle to mobilize the tumour (Figure 2(a)). The internal, external iliac, and common iliac veins were removed with the tumor after dividing the common iliac vein at its junction with inferior vena cava. An en bloc of schwannoma with common iliac, internal iliac and external iliac veins, internal iliac artery, femoral nerve, and obturator nerve and iliopsoas muscle was done (Figure 2(c)). Lower limb vascularity was re-established at the end of the procedure by reanastomosing the cut external iliac artery (Figure 2(b)) and omental flap was wrapped around the anastomosed artery. Ureters, ovaries with its supplying vessels, uterus, and bladder were preserved. The final histopathology was reported as ancient schwannoma (Figure 3).

Postoperatively patient had uneventful recovery. She had patchy sensory loss, foot drop, and weakness of quadriceps muscles. She was rehabilitated with a foot drop splint and active physiotherapy. At last follow-up, she was able to walk without support. Two years after surgery patient is without any evidence of disease.

## 3. Discussion

Ancient schwannomas are rare tumours originating from Schwann cells in the peripheral nerve sheath. Up to 20% of cases are associated with Neurofibromatosis Type 1 [1]. They usually arise in females between the ages of 20 and 50 years [2]. Retroperitoneal schwannomas are rare and account for 0.7% to 5% of these tumors [1]. The microscopic appearance of schwannoma is composed of spindle cell proliferation with hypercellular (Antoni A) and hypocellular (Antoni B) areas [1]. The type A areas often show spindle cells arranged in a palisading fashion or in an organoid pattern called Verocay bodies. Sometimes large nodular masses of collagen with radiating edges are seen which are designated as “amianthoid fibres.” The term “ancient” was used as a description for the degenerative changes apparent on microscopy like marked nuclear atypia where the Schwann cell nuclei are large, hyperchromatic, and multilobed but lack mitotic figures. The tumor often reveals cyst formation, hemorrhage, calcification and hyalinization. These degenerative changes are thought to be due to the increasing tumor size causing vascular insufficiency.

Even though surgical excision is the treatment of choice, the surgeon should be aware of nerve involvement which can cause disabling neurologic deficits. Though the neoplasms are mostly asymptomatic and may be found incidentally on examination or imaging, occasionally they produce pressure effects on surrounding large nerves. The unique problems we encountered in our case were the enormous size, the location of major part of the tumor in the pelvis, the need for fertility preservation, the external iliac vessels stretching over the tumor making mobilization surgically demanding, and the prospects of neurological deficits. In one of the largest series published, the largest size recorded was 22 cm [3]. Another large retroperitoneal schwannoma reported in literature is 28 cm [4].

Computed tomography (CT) and magnetic resonance imaging (MRI) are widely used as imaging techniques in the evaluation of retroperitoneal soft tissue tumors. Imaging characteristic of a schwannoma on CT is that of a well-defined, homogeneous mass with rim enhancement of the fibrous capsule following intravenous contrast administration [5]. The degenerative histological features of ancient schwannomas are evident in their radiographic features as well-circumscribed complex cystic masses with inhomogeneous contrast enhancement. Nonenhancing areas on CT imaging correspond to regions of cystic degeneration, with contrast enhancement seen in surrounding tissues [5].

MRI findings of schwannomas have been reported as masses of low signal intensity on T1-weighted images similar to muscle and high signal intensity on T2-weighted images similar to fat [6, 7]. These findings are characteristic but not specific of schwannomas and have been reported to be present in only 57% of the cases in previous studies, [7] hindering the correct diagnosis. Additionally, the signal intensity on T2-weighted images may vary depending on cell density. Tumors with microscopic findings of hypercellular Antoni type A tissue have intermediate signals, while tumors with Antoni type B tissue have a bright signal on T2-weighted images. MRI with gadolinium enhancement has been advocated as superior to CT in demonstrating tumor cystic degeneration, defining margins, and in some cases identifying the point of neuronal origin [8, 9]. However, radiographic modalities do not differentiate benign from malignant disease unless tumor invasion or metastasis is seen.

2-Deoxy-[(18)F] fluoro-D-glucose(FDG) on PET scanning is of limited value as a preoperative diagnostic imaging technique for the assessment of schwannoma versus sarcoma [10]. High FDG on PET scanning which is used to distinguish malignant from benign tumours is a common feature in benign Schwannoma [11] probably because of high cellularity.

Since in our case the CT scan report was suggestive of malignancy, we decided to do FNAC and core biopsy under ultrasound guidance. This was reported as being benign nerve sheath tumor.

Literature search about surgical management of such tumors revealed a tendency to confuse ancient schwannomas with malignant tumors on imaging and histology [12]. Confusion with malignancy can be avoided by recognizing benign features such preservation of spindle shape with large cohesive aggregates of cells in FNAC and absence of mitosis in spite of worrying nuclear features in an otherwise characteristic histology of schwannoma. Malignant transformation of schwannoma is, in contrast to neurofibroma, exceptionally rare and interestingly, in most of them the malignant component has exhibited an epitheloid morphology [13, 14]. Das Gupta and co-workers noticed the presence of cystic changes in 75% of malignant schwannomas compared to only 6% in benign lesions [15]. Schwannomas react strongly with S-100 protein and immunohistochemistry can be used to aid diagnosis [16]. Flow cytometry assessing DNA ploidy may also help differentiate benign from malignant lesions.

Some workers have argued that since it is a benign disease, even piecemeal excision even with laparoscope is an acceptable alternative [17]. In such situation should the postoperative histology confirm malignancy of the tumor, local recurrence after marginal excision has to be expected in up to 72% of cases, whereas recurrence after resection with a wide surgical margin has been reported in only 11.7% [18]. Therefore many authorities have suggested complete surgical excision as the best management [17].

Hence after deliberations, we decided to treat the mass as potentially malignant and do excision taking into consideration oncological safety. Care must be taken in attempting removal of retroperitoneal and intrapelvic schwannomas. It is of utmost importance to meticulously plan and to involve experts in multiple specialties for optimal management. Sufficient amounts of blood products have to be readily available including fresh frozen plasma and platelets. The anesthetist should be made aware that a high volume blood loss might be encountered.

Carpenter reported one intraoperative death related to uncontrollable hemorrhage from severing the right common iliac artery during a difficult dissection [18]. In a case report by Foote, the attempt to excise a large retroperitoneal schwannoma was abandoned because of the danger of uncontrollable hemorrhage [19].

In our case, both the right external iliac artery and vein were stretched over the tumor. Hence before mobilisation of the tumor, vascular clamps were applied and the artery was cut in the middle. This made mobilisation possible without a catastrophic hemorrhage. The accompanying vein was ligated at superior and inferior edges of the tumor. Venous ligation has the added advantage of preventing a pulmonary embolism in the postoperative phase, due to a preexisting thrombus in the affected lower limb. It is highly desirable to do a preoperative angiogram or MR angiogram to document collateral circulation. In situations where a vascular surgeon is not available, the ligation of major vessel supplying the limb might be the only alternative left. The ligation of internal iliac artery not only helped in mobilizing the tumor, but also decreased the tumor vascularity considerably. Preoperative radiologic guided obliteration of anterior division of internal iliac arteries has been shown to decrease the vascularity of such tumors. Haemostasis can be difficult especially in presacral schwannomas where there is involvement of presacral venous plexus. The vascular surgeon's expertise was a key event in the successful outcome of the surgery. Bull dog clamps are to be considered as an essential instrument in undertaking such surgeries. The end-end anastomosis of external iliac artery was done with No.7.0 Prolene. Postoperative blowouts at the anastomotic site have been reported. The wrapping of omental flap around the anastomosis is a simple but important aspect of surgery that may prevent catastrophic blowouts. Total blood loss was 2.5L. The femoral and obturator nerves were sacrificed as they were found to be going through the tumor. This, however, did not cause significant morbidity other than quadriceps weakness and patchy sensory loss in the right leg. Patient also developed foot drop probably due to nerve root damage. Since neurological deficits are to be anticipated, proper preoperative counseling and preparation of the patient are extremely important before undertaking such surgeries.

## 4. Conclusion

We described a huge retroperitoneal abdominopelvic tumor, with radiological features of malignancy, treated by radical excision with organ preservation and minimal long-standing sequelae. Surgical management of this tumor gives insights into complexities that are likely to be encountered while managing such a case. The neurovascular supply of lower limbs has to be given due consideration and vascular surgery planned so that on-table surprises can be avoided.
